# Modular Protein Ligation: A New Paradigm as a Reagent Platform for Pre-Clinical Drug Discovery

**DOI:** 10.1038/s41598-019-49149-2

**Published:** 2019-09-11

**Authors:** Rosalie Matico, Lawrence M. Szewczuk, Beth Pietrak, Stephanie Chen, Ed Dul, William G. Bonnette, Derrick W. Meinhold, Geoffrey Quinque, Rachel Totoritis, Tia Lewis, Maggie Grimes, Daniel Fornwald, Patricia M. McCormick, Michael Schaber, Yong Jiang, Randy Bledsoe, Marc A. Holbert

**Affiliations:** 1 GlaxoSmithKline, 1250S Collegeville Rd., Collegeville, Pa 19426 USA; 2 Janssen Pharmaceutical Companies of Johnson and Johnson, 1400 McKean Rd., Springhouse, Pa 19477 USA

**Keywords:** Drug screening, Proteins

## Abstract

Significant resource is spent by drug discovery project teams to generate numerous, yet unique target constructs for the multiple platforms used to drive drug discovery programs including: functional assays, biophysical studies, structural biology, and biochemical high throughput screening campaigns. To improve this process, we developed Modular Protein Ligation (MPL), a combinatorial reagent platform utilizing Expressed Protein Ligation to site-specifically label proteins at the C-terminus with a variety of cysteine-lysine dipeptide conjugates. Historically, such proteins have been chemically labeled non-specifically through surface amino acids. To demonstrate the feasibility of this approach, we first applied MPL to proteins of varying size in different target classes using different recombinant protein expression systems, which were then evaluated in several different downstream assays. A key advantage to the implementation of this paradigm is that one construct can generate multiple final products, significantly streamlining the reagent generation for multiple early drug discovery project teams.

## Introduction

**Figure 1:**
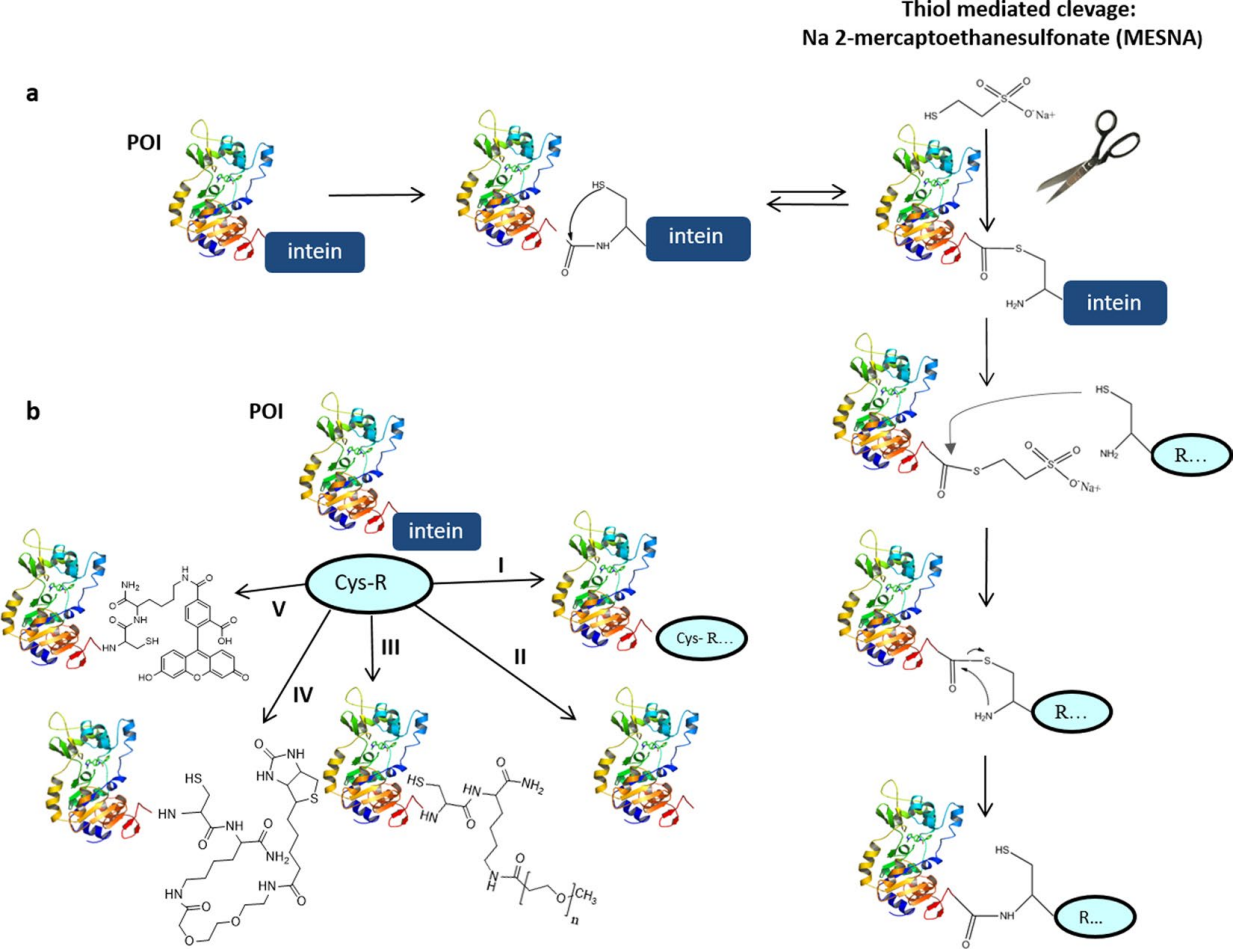
Development of Modular Protein Ligation. (**a**) Conceptualization of EPL. The protein of interest (POI) is expressed as C-terminal intein fusion. The genetically modified intein (catalytic Asn mutated to Ala to block final excision step) catalyzes an N-S acyl shift, which in the presence of excess thiol, results in the release of the intein and the formation of a thioester at the C-term of the POI. The thioester undergoes attack from an N-term cysteine linked to a respective functional moiety (termed R group) and undergoes a spontaneous S-N acyl shift to form a stable peptide bond between the POI and the cysteine-containing peptide/protein fragment. (**b**) Conceptualization of MPL. Moving clockwise from the POI-intein fusion: (I) Cys-R, where R represents a functional moiety such as lanthanide binding peptide, 10xHis, dual STREP, etc.…, (II) free C-terminus of POI generated in presence of excess DTT, (III) POI with Cys-[K-(PEG)_n_], (IV) POI with Cys-[K-PEG2-Biotin], or (V) POI with Cys-[K-Fluorescein].

Inteins are the protein equivalent to introns in RNA. As internal sequences in a precursor protein they catalyze a multi-step biochemical reaction that results in their excision from a precursor sequence and ligation of two extein flanking sequences to form a new functional protein. Inteins were initially discovered in the early 1990s in lower organisms. Their potential for protein engineering was quickly realized, which resulted in the subsequent identification of many additional inteins in the coming years. Inteins have been exploited for protein engineering either as split inteins known as protein trans-splicing (PTS) or through expressed protein ligation (EPL), a technique that utilizes genetically engineered inteins to join two protein (peptide) fragments via a native peptide bond^1–4^. In this study we focused on the latter technique, EPL. Historically EPL has been used to install various site-specific protein modifications to enable detailed analysis of signaling pathways, enzyme regulation, and protein-protein interactions^5,6^. In addition, this technique has been utilized for segmented isotopic labeling for protein NMR^7,8^ or as a cleavable purification tag^3,9^. Genetically engineered versions of *Sce* VMA1 and *Mxe* GyrA are commonly used for C-term labeling on proteins of interest (POI)^10,11^. The C-term Asn in these inteins has been mutated to Ala blocking the final excision step. This allows the inteins to catalyze a N-S acyl shift at the junction between the C-term of the POI and the N-term cysteine residue of the intein (Fig. 1a) but progress no further. A normal peptide bond can reform unless an external thiol is added to form a more stable thioester. Upon addition of the thiol, the intein is released from the C-term of the POI, and this new thioester is susceptible to attack from an N-term cysteine linked to a respective functional moiety (termed R group) via an amide bond. The resulting thioester undergoes a spontaneous S-N acyl shift to form a stable peptide bond between the POI and the newly attached cysteine peptide/protein fragment (Fig. 1a)^12^.

A standard model for reagent generation has been the creation of multiple constructs for different end uses, with the potential for redesign through successive rounds of trial and error. MPL has the potential to provide a combinatorial platform for one construct - multiple products to support different screening/biophysics platforms, thus reducing timelines for hit identification and subsequent hit qualification. To lay the foundation for this labeling platform, we developed a library of cys-lys dipeptides chemically labeled on the ε-amino group of the lysine and cys-peptide tags. Proteins of various sizes in different target classes were evaluated, with most of the protein targets expressed in *E coli*. The C-terminal residue of these protein targets is critical for optimal ligation; often an alanine is introduced to improve ligation efficiencies. We demonstrated the feasibility of recombinant expression for the intein fusion proteins in both insect and mammalian expression systems including the secretion of target proteins into the growth media.

## Results

**Figure 2:**
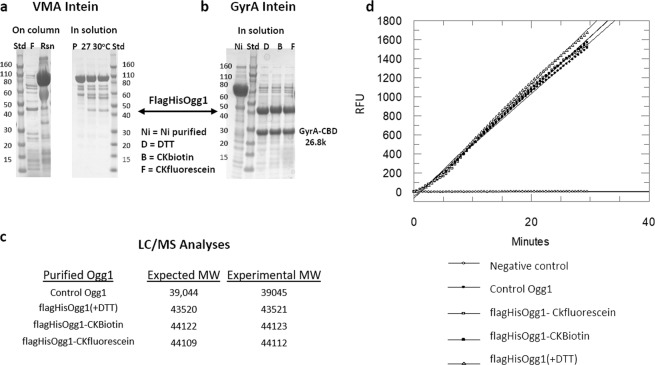
Choice of intein impacts ligation. (**a**) FlagHisOgg1VMA-CBD Coomassie stained SDS-PAGE comparison of “On column” to “In solution ligations.” “On column” was tested with 2 mM C[K-fluorescein] (gel column F) on chitin resin (marked “Rsn” on gel) for 20 hr at room temperature. “In solution” ligations tested with Ni purified FlagHisOgg1VMA-CBD (marked P on gel) in 2 mM cysteine, 0.2 M MESNA at varying temperatures; 40 hr timepoint shown. (**b**) Coomassie stained SDS-PAGE of FlagHisOgg1GyrA-CBD in solution ligations, where DTT (marked D on gel) was used to generate FlagHisOgg1 with no C-term label for 20 hours at room temperature. “In solution” ligations were done in the presence of 2 mM Cys-Lys dipeptides and 0.2 M MESNA at room temperature overnight. (**c**) LC/MS confirmation of successful ligation and non-labeled control. (**d**) Catalytic activity of various Ogg1 constructs was assayed.

As demonstrated in Table 1, irrespective of class, most targets can be successfully labeled through expressed protein ligation. However, the choice of intein, as observed with Ogg1 in Fig. 2, can impact the ligation process. The inefficiencies in the EPL reaction observed with the flagHisOgg1VMA1-CBD construct were not overcome with scouting for optimal reaction conditions. However, switching the VMA intein to the GyrA intein improved ligation efficiencies to >95% (Fig. 2b). LC/MS confirmed the successful ligation of the labeled di-peptides, as well as the non-labeled control generated by classic DTT mediated cleavage (Fig. 2c). Activity analysis of the final purified products showed no differences in catalysis between the EPL generated Ogg1 compared to the control Ogg1 (Fig. 2d).

**Table 1:** Summary of MPL generated protein reagents across various target classes.

E. coli Expression - Intracellular Proteins	Modification	Potential End Use	Yield Final Product
IDO1 (Uniprot P14902)	Biotin	ELT/SPR	0.24 mg/g
	Cy5	MST/cmpd aggr.	~0.2 mg/g
	none	Activity	0.6 mg/g
murine Keap1(Uniprot Q9Z2X8)	lanthanide	NMR	> 5 mg/g
	Biotin	ELT/SPR	~1 mg/g
	10His	ELT/SPR	~1 mg/g
	Fluorescein	MST/cmpd aggr.	~1.5 mg/g
E. coli LpxC (Uniprot P0A725)	Biotin	ELT/SPR	> 0.4 mg/g
	none	Activity	~0.4 mg/g
P. aeruginosa LpxC (Uniprot P47205)	dual Strep	SPR	~0.5 mg/g
	Biotin	ELT/SPR	~0.5 mg/g
Ogg1 expressed with GyrA (Uniprot O15527)	Fluorescein	MST/cmpd aggr.	2.25 mg/g
	Biotin	ELT/SPR	2.6 mg/g
	None	unlabeled control	2.3 mg/g
Sirt1(1–747) (Uniprot Q96EB6)	Biotin	ELT/SPR	> 0.14 mg/g
	Fluorescein	MST/cmpd aggr.	> 0.14 mg/g
STING (149–379) H232R (Uniprot Q86WV6)	Biotin	ELT/SPR	> 0.2 mg/g
	Fluorescein	MST/cmpd aggr.	> 0.1 mg/g
**Baculoviral Expression - Intracellular Protein**			
Sirt1(183–664) expressed with VMA	Biotin	ELT/SPR	1.5 mg/g
	Fluorescein	MST/cmpd aggr.	1.0 mg/g
Sirt1(183–664) expressed with GyrA	Biotin	ELT/SPR	1.3 mg/g
	Fluorescein	MST/cmpd aggr.	1.3 mg/g
RIPK1 (Uniprot Q13546)	Biotin	ELT/SPR	~0.01 mg/g
	lanthanide	NMR	~0.04 mg/g
	none	control	~0.01 mg/g
**Baculoviral Expression – Secreted**			
TFR1 (L122-F760) (Uniprot P02786)	Biotin	SPR	0.05–0.16 mg/L
	Fluorescein	MST/cmpd aggr.	0.14 mg/L
**Mammalian Expression of Secreted Protein**			
soluble CD73 (Uniprot P21589)	Fluorescein	MST/cmpd aggr.	> 5.0 mg/L Medium
	Biotin	ELT/SPR	> 5.0 mg/L
	Dansyl	Trp Fl	Trp Fl
	Cy5	MST	MST
	10His	SPR	SPR
	None	Activity	~1.0 mg/L
IGD domain of aggrecan (Uniprot P16112)	Biotin	SPR	~1.0 mg/L

**Figure 3:**
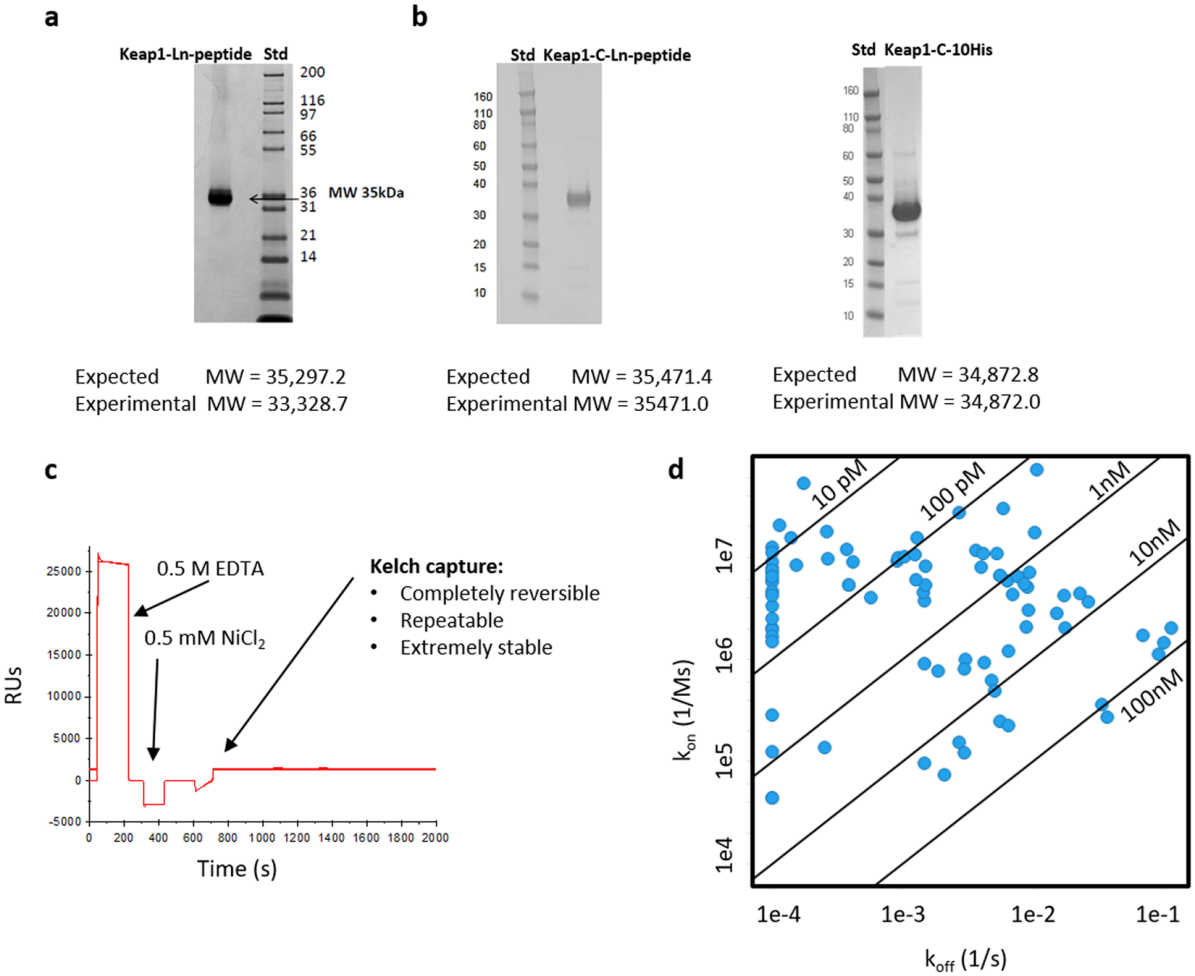
EPL Enables Alternative strategy for C-term peptide labeling. (**a**) SDS-PAGE and LC/MS analysis of murine Keap1 (322–624)-lanthanide generated via classical construct design with lanthanide peptide engineered into expression construct. (**b**) SDS-PAGE and LC/MS analyses of murine Keap1(322–624) with C-term lanthanide peptide or 10His peptide added via EPL (**c**) Regeneration of SPR chip enabled with murine Keap1(322–624)-Cys10His (**d**) High throughput binding kinetics of small molecules enabled by SPR; each blue circle represents a distinct compound. Several compounds had k_off_ rates that were measured to be less than 1e-4, hence they could not be reliably fit and appear “pegged” to the y-axis.

Hit discovery platforms are supported with chemical labels as well as specific peptide tags. Typically, peptide tags are engineered into the desired expression construct. EPL provides a path to streamline these reagents in a timely manner when classic expression and purification can be challenging. As demonstrated in Fig. 3, the Kelch domain of murine Keap1 was expressed with a C-terminal lanthanide (Ln) peptide^13,14^, HisAviThr murine KEAP1(322–624)-Ln-peptide. The final purified murine KEAP1(322–624)-Ln-peptide was truncated and did not include the lanthanide peptide based on LC/MS analysis (Fig. 3a). Generation of the murine HisThrKeap1(322–624)GyrA-CBD enabled the production of intact murine Keap1 (322–624)-C-Ln-peptide as confirmed by LC/MS (Fig. 3b). This new construct streamlined the generation of murine Keap1 (322–624)-C-10His for SPR studies. Initial SPR assay development for the Kelch domain utilized traditional amine-coupling methods. This SPR assay format was sufficient for the initial hit to lead medicinal chemistry efforts, but as the program transitioned to lead optimization and the compounds became more potent, the initial SPR assay became limited. The throughput of the initial SPR assay decreased as lead optimization of the initial hits increased the binding affinity of the compounds, while also optimizing for slow dissociation rates. Regeneration conditions were unacceptable for Kelch protein stability, requiring a new surface for each SPR experiment. However, the successful generation of murine Keap1(322–624)-C-10His via EPL enabled SPR surface regeneration (Fig. 3c) resulting in a ~10-fold increase in assay throughput (Fig. 3d), and significant cost savings on sensor chips. Murine Keap1(322–624)GyrA-CBD, a single construct, was used to generate 4 different murine Keap1(322–624) proteins containing one of the following C-terminal modifications: biotin, lanthanide binding peptide, fluorescein, or a 10xHis affinity tag.

**Figure 4:**
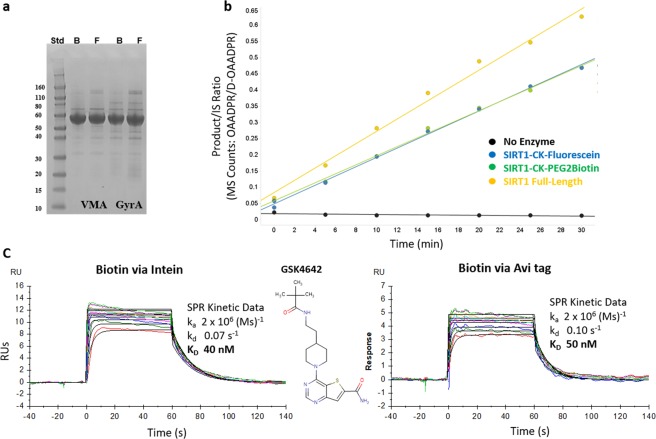
Intracellular Baculoviral expression of mini Sirt1. (**a**) SDS-PAGE comparison of final products labeled with either CK-biotin (marked B on gel) or CK-fluorescein (marked F on gel) from the flagHisTevSirt1(183–664)VMA-CBD (marked VMA) or flagHisTevSirt1(183–664)GyrA-CBD (marked GyrA). (**b**) Activity comparison of intein labeled mini-Sirt1 vs full-length Sirt1. (**c**) SPR comparison of mini-Sirt1 labeled via Bir A versus EPL for binding to GSK4642.

To demonstrate the utility of MPL in insect cells, we compared the intracellular baculoviral expression of Sirt1(183–664; referred to as mini-Sirt1), fused with either the VMA or GyrA inteins. Unlike Ogg1, the ligation of mini-Sirt1 with both inteins was highly efficient. Final product yields (Table 1) and purity (Fig. 4a) were equivalent with both inteins and the ligation peptides used. Activity analysis confirmed that the C-term labeling by EPL did not impact the deacetylase activity of mini-Sirt1 compared to full-length Sirt1 under the given experimental conditions (Fig. 4b). SPR studies demonstrated that similar binding kinetics could be measured for 3 compounds for both the EPL biotin labeled mini-Sirt1 and mini-Sirt1 biotin labeled via the Avi-tag (Fig. 4c; data shown for one compound). In parallel, a fluorescein labeled mini-Sirt1 was generated to evaluate on target compound aggregation^15^.

**Figure 5:**
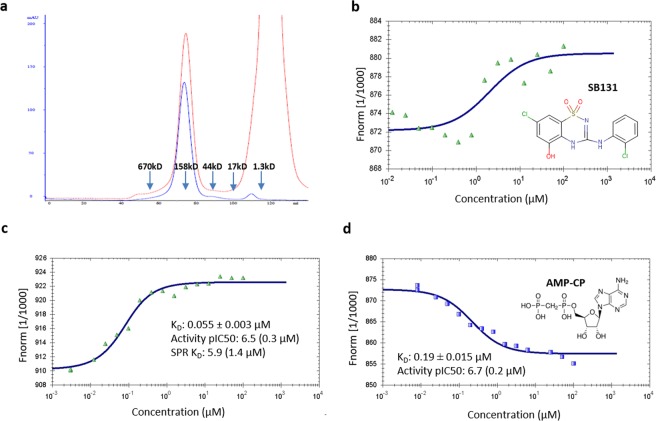
Mammalian expression of soluble CD73 (**a**) Superdex 200 comparison of final CD73 labeled with either CK-biotin (blue trace at 280 nm) or CK-fluorescein (red hatched trace at 495 nm) with migration of MW standards indicated by arrows (**b**) MST of SB131 binding to CD73 NHS labeled^28^ (**c**) MST pf CD73 EPL fluorescein labeled (**d**) MST of AMP-CP binding to CD73 EPL fluorescein labeled.

The versatility of MPL was further expanded with the mammalian secreted expression of sCD73, a hydrolase that forms a non-covalent dimer, containing 4 intramolecular disulfide bonds per chain. The previous reported use of FcGyrA from baculoviral secretion to form Fc-small molecule conjugates informed on our selection of GyrA for all tested secretion systems in this study^16^. sCD73 was transiently over-expressed in HEK293 cells using a BacMam encoding a GyrA-CBD fusion protein. The GyrA-CBD did not negatively impact the ability of sCD73 to be secreted into the growth media. The EPL reaction for the intracellular expressed proteins was typically done in the presence of 200 mM MESNA, but this proved detrimental to sCD73 (data not shown). Therefore, the MESNA concentration was reduced to 36 mM to generate biotin and fluorescein labeled sCD73. The labeled CD73 protein(s) migrated as the expected dimer by SEC using a Superdex 200 column, with no observed aggregate peak (Fig. 5a). The EPL fluorescein labeled sCD73 was the superior final product for MST analysis when compared to the NHS-Red labeled sCD73, as evidenced by the improved quality of the binding isotherm and subsequent K_d_ determination (Fig. 5b,c). Furthermore, the fluorescein labeled sCD73 was used to determine the dissociation constant (K_d_) for an analogue of CD73’s AMP substrate (AMP-CP; Fig. 5d). Again, a single sCD73 (Table 1) intein construct was successfully modified with five different ligands, enabling multiple qualification assays for target MOA, that proved challenging to produce using traditional recombinant protein production methods.

The use of inteins and subsequent ligations can both positively and negatively affect final recombinant protein yields when compared to traditional tagging strategies. For example, if we did not use MPL, we were unable to express and purify an intact, recombinant Kelch domain of murine Keap1 fused to the lanthanide peptide. Expression and purification of Ogg1 in bacteria and mini-Sirt1 using baculovirus had similar protein yields when comparing MPL to more traditional recombinant protein production strategies. We were able to obtain yields of 2 mg/g cell paste for FlagHis6-Ogg1 expressed in *E*. *coli*. (data not shown) compared to yields of 2.2–2.6 mg/g cell paste using inteins (Table 1). Comparable yields were also seen with mini Sirt1: 1–1.5 mg/g cell paste using inteins (Table 1) and 2 mg/g cell paste with no intein^17^. Two examples of inteins negatively affecting final protein yields were our experience with full-length Sirt1^18^ in bacteria and secreted CD73 in a mammalian system. The use of inteins decreased final protein yields by 10-fold in both cases. Hence, the use of the MPL platform is broadly enabling and can be extended across most recombinant expression systems (bacteria, insect, and mammalian), and can even be engineered for secretion into the growth media enabling biopharmaceutical and other higher yield applications.

## Discussion

In drug discovery the paradigm has been to generate multiple constructs in the evaluation of multiple hit ID and hit qualification platforms. There is significant trial and error inherent in this process. In addition to multiple construct designs, nonspecific chemical labeling to support these platforms has significant potential to impact protein yield and quality. In this study, we introduce MPL, a powerful, combinatorial approach to quickly and site specifically functionalize protein targets for multiple downstream applications using a single construct. We illustrate the utility of this approach by using 2 inteins (*GyrA* and *VMA*) to introduce 7 different modifications to 11 different protein targets, resulting in 34 different functionalized proteins of interest. These functionalized proteins were then tested in 8 downstream applications including: biophysical assays (SPR, MST, and compound aggregation), structural biology (protein NMR), biochemical activity characterization, and hit identification (ELT). Compared to traditional approaches for recombinant protein generation, our intein-based MPL platform resulted in the reduction in the number of constructs generated by half, in addition to significant cost and time savings.

This MPL platform of one construct to generate multiple final products to enable drug discovery efforts (Table 1), provides a new, broadly applicable paradigm for combinatorial reagent generation, with plenty of room for continued growth. Expansion of the functionalized di-peptide library presented here, will greatly increase the number of downstream applications and assays that can be used with this approach. Furthermore, during the preparation of this manuscript, Dempsey *et al*. published a simple method for converting NHS-esters into thioesters which can be used to stoichiometrically modify N-Cys containing proteins^19^. This technique enables N-term labeling of targets sensitive to C-term modifications and can further expand the scope of MPL to include dual modifications at the N- and C- termini. In this scenario, using the 7 modifications explored in this manuscript, a single N-Cys/C-intein construct could yield 56 different protein reagents bearing modifications at either the N-term, C-term, or a combination of both. Dual modification of a single protein, for example with a FRET pair, will enable additional downstream assays, specifically those tailored toward protein dynamics and conformational change.

## Methods

### Recombinant Ogg1 expression and purification

Full-length human Ogg1, transcript variant 1a (8-oxoguanine DNA glycosylase (OGG1)), was sub cloned into pDEST-T7flagHis6-ST from pENTRDTEVhumanOgg1v1a_FL using an LR clonase reaction. pDEST-T7flagHis6-SThumanOgg1v1a_FL was freshly transformed into BL21(DE3) */pRR692, then scaled to 1 L in LB Broth with 1% glucose, 100ug/ml carbenicillin and 25ug/ml chloramphenicol. The 1 L culture was then inoculated to 20 L in 2xYT broth with 1X Vogel-Bonner salts in 1% glucose with 100ug/ml carbenicillin, and 25ug/ml chloramphenicol at 37 °C and induced at OD_600nm = _5.27 for 20 h at 15 °C with 0.2 mM IPTG. FlagHisTEVOgg1 was released from E. coli cell paste by pressure drop lysis at 15,000 psi and treated with Benzonase. The flagHisTEVOgg1 was captured onto Ni-NTA agarose from cell lysate supernatant. After an extensive wash flagHisTEVOgg1v1a was eluted with 250 mM imidazole, digested with TEV protease, desalted on Sephadex G25 prior to high resolution chromatography on Mono S at pH = 6.

### Recombinant Ogg1 expression, purification, and ligation(s)

DNA encoding full length human Ogg1 with a tobacco etch virus (TEV) cleavage site on the N-term and a C-terminal Ala followed by the VMA intein and chitin binding domain (CBD) was synthesized at Genscript and inserted into pENTR1a vector. pDEST T7 flagHisTEVOgg1VMA-CBD was generated through a GATEWAY LR reaction of the pENTR1a TEVOgg1VMA-CBD with pDEST T7 flagHis6 and transformed into pRR692/BL21STAR(DE3). Freshly transformed cells were scaled in 2X YT Broth with Ampicillin (100ug/ml) and Chloramphenicol (35ug/ml) and induced overnight with 0.1 mM IPTG at 20 °C. FlagHisTEVOgg1VMA-CBD was released from E. coli cell paste by two passes through a Microfluidizer at 10,000 psi, and captured onto chitin resin (New England BioLabs) from clarified lysate supernatant. Chitin resin was thoroughly washed prior to the overnight room temperature incubation with 2 mM C[K-fluorescein] in 200 mM MESNA 25 mM HEPES 0.25 M NaCl 0.1 mM TCEP pH = 7.4. FlagHisTEVOgg1VMA-CBD in the unbound from the overloaded chitin resin was captured by Ni-NTA Agarose (Invitrogen). After an extensive wash flagHisTEVOgg1VMA-CBD was eluted with 250 mM imidazole, concentrated, and sized on Superdex 200 into 25 mM HEPES 0.25 M NaCl 0.1 mM TCEP pH = 7.4. Ligation to free cysteine was monitored after a 2-fold dilution into 4 mM cysteine 400 mM MESNA 25 mM HEPES 0.25 M NaCl pH = 7.4 for up to 69 hr at 27 °C, 30 °C, and 34 °C.

DNA encoding human flagHisTEVOgg1GyrA-CBD with an alanine inserted between Ogg1 and GyrA was synthesized at Genscript and cloned into pET24b. The pET24b flagHisTEVOgg1GyrA-CBD was freshly transformed into BL21star(DE3) T5R, scaled at 37 °C in LB broth with 1% glucose, and induced for 20 h at 15 °C with 0.5 mM IPTG with kanamycin (50ug/ml) and chloramphenicol (25ug/ml). FlagHisTEVOgg1GyrA-CBD was released from E. coli cell paste by two passes through a Microfluidizer at 10,000 psi, captured onto Ni-NTA agarose from clarified lysate supernatant, and eluted with 250 mM imidazole. The Ni-eluate was dialyzed against 25 mM HEPES 0.25 M NaCl 0.1 mM TCEP pH = 7.4 and incubated overnight at room temperature with 2 mM labeled dipeptides (C[K-PEG2-Biotin] or C[K-fluorescein]; 21^st^ Century Peptides) in 25 mM HEPES 0.25 M NaCl 0.1 mM TCEP 0.2 M MESNA pH~7.4, or 40 mM DTT in 25 mM HEPES 0.25 M NaCl pH~ 7.4. The ligations and C-term cleavage were extensively dialyzed and flowed through chitin resin to remove GyrA-CBD.

### Ogg1 activity assay

Ogg1 catalytic activity^20^ was tested at 50 nM purified Ogg1, 100 nM 5′-FAM-ACT-8-OxoGuanine-AAGCGCGCACGCCATGTCGACGCGCTTCAGT-DAB-3′ oligo (Integrated DNA Technologies), with and without 100 uM 8-Bromo-Guanine in 25 mM HEPES pH = 7.5 50 mM KCl 1 mM EDTA 1 mM CHAPS 1 mM DTT 0.01% nuclease free BSA with 30 seconds reads over a 30-minute time course using a Viewlux Imager for fluorescence intensity (ex = 480 nm; em = 540 nm; mirror FITC Dichroic).

### Keap1 expression, purification, and ligation(s)

The synthesized DNA of the lanthanide peptide^13,14^ was inserted between the NotI and XhoI sites of pET15b His avi murine KEAP1(322–624)^21^. pET15b His avi murine KEAP1(322–624) lanthanide was freshly transformed BL21star(DE3) T5R, scaled at 37 °C in LB broth with 1% glucose, and induced for 20 h at 15 °C with 0.5 mM IPTG. HisAviThrombin(Thr)murine KEAP1(322–624) lanthanide was released from the E. coli cell paste by three passes through a Microfluidizer at 12,000 psi, and captured onto Ni NTA Superflow (Qiagen). Murine KEAP1(322–624) lanthanide was released from the Ni NTA Superflow by thrombin, then sized on Superdex 200.

DNA encoding murine HisThr Keap1(322–624)GyrA-CBD with an alanine inserted at the C-term of Keap1(322–624) prior to the GyrA was synthesized at Genscript and inserted into pET24b. The pET24b murine HisThrKeap1(322–624)GyrA-CBD was freshly transformed into BL21star(DE3) T5R, scaled at 37 °C in LB broth with 1% glucose, and induced for 20 h at 16 °C with 0.5 mM IPTG. Murine HisThrKeap1(322–624)GyrA-CBD was released from the E. coli cell paste by two passes through a Microfluidizer at 12,000 psi, and captured onto chitin resin from clarified lysate supernatant. Washed chitin beads were incubated with 0.36 mM to 2 mM peptides CYIDTNNDGWYEGDELLA-amide (C-ln-peptide) or CHHHHHHHHHHH (C-10His), respectively (21^st^ Century Peptides) in 36 mM to 80 mM MESNA at pH = 7.5 for 23 hours at room temperature. murine HisThrKeap1(322–624)peptide in the chitin unbounds was dialyzed to reduce MESNA, digested with thrombin to remove his-tag, and sized on Superdex 200 for the murine Keap1(322–624)C-ln-peptide and Superdex 75 for murine Keap1(322–624)C-10His.

### SPR for Keap1

SPR of Keap1 was performed in HBSN buffer with 0.005% P20, 1% DMSO, and 0.5 mM TCEP. An NTA chip (GE Healthcare) was conditioned with 0.5 M EDTA (60 s) followed by 10 mM NaOH (30 s). The NTA surface was pre-equilibrated with 0.5 mM NiCl2 (15 s) and Keap1 was captured by injecting a 100 nM solution of murine Keap1(322–624)C-10His (30 s). Compound titrations were run using a 5 point 3-fold dilution single cycle kinetics method with a top concentration of 100 nM. Association and dissociation times were set to 60 and 500 s respectively. Prior to titrating compounds, a series of buffer injections were carried out to serve as blanks. The flowrates for conditioning, Keap1 capture, and compound titrations were 30, 10, and 50 uL/min respectively. To ensure an un-complexed target at the start of each titration, following each compound titration, Keap1 was removed from the surface using EDTA, the surface was pre-treated with NiCl2, and Keap1 was re-captured as described above.

### IDO1 expression, purification, cleavage and ligation

DNA encoding human IDO1 with a TEV cleavable N-term 6Hisflag tag and C-term alanine followed by GyrA-CBD was synthesized at Genscript and inserted into pet24b vector. The pET24b-HisflagTEVIDO1GyrA-CBD was freshly transformed into BL21(DE3)*, cell growth was carried out at 37 °C in E.coli production broth (LB Broth or Terrific Broth Complete) with carbon source (1% glucose/glycerol), 50 µg/ml kanamycin, and induced for 20 h at 15 °C with 0.5 mM IPTG in broth with 1% glycerol, 50 µg/ml kanamycin, 100 µM Hemin, and 100 µM FeCl3. HisflagTEVIDO1GyrA-CBD was released from E. coli cell paste by two passes through a Microfluidizer at 12,000 psi, and captured onto chitin resin from clarified lysate supernatant. The Hisflag was removed by on column TEV cleavage followed by ligation with 2 mM labeled di-peptides (C[K-PEG2-Biotin], C[K-Cy5] 21^st^ Century Peptides) in 150–200 mM MESNA in 25 mM HEPES 0.25 M NaCl 0.1 mM TCEP pH = 7.4 or cleavage in the buffered MESNA without di-peptide. Ligated and cleaved IDO1 was concentrated and diafiltered against 25 mM Tris pH 8, 0.1 mM TCEP at room temperature prior to processing on 5 ml QHP HiTrap (GE Healthcare). IDO1 containing Q pools were incubated with 0.2 mM Hemin for 2 hr at 4 °C prior to sizing on Superdex200.

### Lpxc expression, purification, and ligations

E. coli LpxC(1–300)P300AGyrA-CBDflagHis and Pseudomonas LpxC(1–299)C40S,A289D,P299A GyrA-CBDflagHis were synthesized at Genscript and cloned into pET24. Plasmids were transformed into competent host strain BL21(DE3)*/PlysS Invitrogen, seed culture grown at 37 °C in LB broth with 1% glucose, kanamycin (50ug/ml) and chloramphenicol (34ug/ml), scaled in Terrific Broth with 1%glycerol, kanamycin (50ug/ml), and chloramphenicol (34ug/ml) and induced for 20 h at 15 °C with 0.5 mM IPTG. LpxCGyrA-CBDflagHis constructs were released from E. coli cell paste by two passes through a Microfluidizer at 12,000 psi, and captured onto chitin resin from clarified lysate supernatant. Chitin resins were thoroughly washed prior to the addition of 2 mM peptides (C[K-PEG2-Biotin], CKKGSAWSHPQFEKGGGSGGGSGGSAWSHPQF EK-amide (dualStrep); 21^st^ Century Peptides) in 200 mM MESNA 25 mM HEPES 0.15 M NaCl 0.1 mM TCEP pH = 7.4 or 50 mM DTT in 25 mM HEPES 0.15 M NaCl 0.1 mM TCEP pH = 7.4. The labeled and unlabeled LpxC released from chitin resins were sized on Superdex 200.

### Sirt1(1–747), Sting (149–379)h232r, expression, purification, and ligations

DNA encoding human full-length human Sirt1 and human STING(149–379)H232R^22^ with a CM N-term linker was synthesized at Genscript with a TEV cleavable site on the N-term and C-term alanine followed by VMA-CBD. The synthesized DNA was inserted into pENTR1a vector. Plasmids were generated through a GATEWAY LR reaction of the pENTR1a vectors with pDEST T7 FLAG His6 and transformed into pRR692/BL21STAR(DE3). Freshly transformed cells were scaled in 2X YT Broth with Ampicillin (100ug/ml) and Chloramphenicol (35ug/ml) and induced overnight with 0.1 mM IPTG at 20 °C. FlagHisTEVSIRT1(1–747)VMA1-CBD and FlagHisTEVSTING(149–379)H232RVMA1-CBD were released from E. coli cell paste by two passes through a Microfluidizer at 10,000 psi, and captured onto chitin resin from clarified lysate supernatant. Chitin resins were thoroughly washed prior to the addition of 2 mM labeled di-peptides in 200 mM MESNA 25 mM HEPES 0.25 M NaCl 0.1 mM TCEP pH = 7.4. Ligations progressed at room temperature for ~24 hr, chitin unbounds were collected, concentrated, and sized on Superdex 200.

### Sirt1(1–747) control protein expression and purification

HisSirT1(1–747) was generated with modifications to previously described protocol^18^. Expression was carried out in SuperBroth with induction at 15 °C overnight in 0.5 mM IPTG. HisSirT1(1–747) was released by 15000 psi pressure drop lysis in 50 mM Tris HCL pH7.5 250 mM NaCl 25 mM imidazole 0.1mMTCEP, captured onto Ni-NTA agarose and eluted step wise with 50 mM and 250 mM imidazole. HisSirT1(1–747) fractions were pooled, concentrated, and sized on Superdex 200. HisSirT1(1–747) was then captured onto Mono Q, step washed to 0.2 M NaCl and eluted during a linear gradient to 0.35 M NaCl in 30 mM Tris HCl pH7.45 0.1 mM TCEP.

### Sirt1(183–664) expression, purification, and ligation(s)

E. coli optimized Sirt1 aa183–664 (miniSirt1)^17^ was PCR amplified out of pET24aH6tevG-optSirt1(1–747) using primers to add an N term HisTEV tag and C term Avi tag. pDONR221. HisTEVSirt1(183–664)Avi was moved into pDEST.T7 by Gateway LR reaction. pDEST T7 HisTEVSirt1(183–664)Avi was transformed into Bl21*(DE3) + pGro7. Cultures were grown at 30 °C; 0.2% arabinose was added for 1 hr at 30 °C followed by induction with 0.5 mM IPTG at 15 °C for 28hrs. HisTEVSirt1(183–664)Avi was released from E. coli cells by pressure drop lysis at 10,000 psi, captured onto Ni-NTA agarose, eluted with 50 mM imidazole, then biotinylated at 30:1 (Sirt1:BirA) for 30 min at 30 °C with 50 μM D-Biotin in 50 mM Tris 80 mM ATP pH 8.0, followed by sizing on Superdex 200^23^.

DNA encoding Sirt1(183–664) with a dual N-term flagHis tag followed by a TEV cleavage site and C-term alanine followed by GyrA-CBD or VMA1-CBD was synthesized at Genscript and inserted into pFASTBAC1. Plasmid DNA is transformed into DH10Bac cells, the recombinant Bacmid (white colony) is grown up overnight and the recombinant Bacmid DNA is prepped using Qiagen mini prep kit. SF9 cells are transfected with Bacmid DNA using Fugene transfection reagent to make Baculovirus. Baculoviral infected insect cells were generated and protein was expressed in SF9 cells^24^. All protein purification steps were carried out at 4 °C unless otherwise indicated. FlagHisTEVSirt1(183–664) intein (GyrA or VMA) was released from baculoviral infected SF9 cells by sonication and captured by batch adsorption onto chitin resin from clarified lysate supernatant. The chitin beads were thoroughly washed prior to the addition of 2 mM labeled di-peptides, C[K-PEG2-Biotin] or C[K-fluorescein], in 200 mM MESNA at pH = 7.5. The ligations progressed at room temperature for 24 hr. The flagHisTevSirt1(183–664)-C[K-label] released from chitin was concentrated prior to sizing on Superdex 200.

### Sirt1 activity assay

Sirt1 catalytic activity was measured in a Rapidfire assay as previously described with modifications^18^. Reactions were initiated by mixing 5ul 20 nM Sirt1 in 50 mM HEPES pH 7.5, 150 mM NaCl, 0.05% CHAPS, 100 ug/mL BSA with 5ul 4 uM Ac-RHK-K(Ac)-W-NH_2_ peptide (American Peptide Co), 20 uM NAD (Calbiochem) 4 uM D-OAADPr^16^ in 50 mM HEPES pH 7.5, 150 mM NaCl, 0.05% CHAPS, 100 ug/mL BSA in a 384-well Greiner Polypropylene V-bottom plate. Reaction wells were quenched every 5 minutes for a total of 30 minutes through the addition of 10 uL of a 20x concentration of NAM (10 nM final concentration) in 50 mM HEPES pH 7.5, 150 mM NaCl. 30 uL of water was then added to each well to bring the total well volume to 50 uL. The plate was loaded onto an Agilent RapidFire v. 3.4/Sciex 4000 Q-Trap RF-MS instrument for analysis [C18 SPE cartridge, 0.2% w/v formic acid in 100% water as the aqueous eluent and 1 mM ammonium acetate in 80% acetonitrile/ 20% water as the organic eluent]. For data analysis the extracted ion chromatogram areas of both the OAADPr product and D-OAADPr internal standard^18^ were recorded. The ratio of OAADPr product and D-OAADPr internal standard were plotted at each assay time point to derive the relative reaction rate for each SIRT1 construct.

### SPR for Sirt1

Biotinylated Sirt1 samples were diluted to 45 ug/ml and captured onto neutravidivin coated chips, which were generated by amine coupling neutravidin to a CM5 chip (GE healthcare). Compound binding was measured under the following conditions, HBS-N with 5 uM ZnCl2, 1 mM DTT, 0.005% P20, 1% DMSO, using an 8-point dose response titration with a top concentration of 12.5 uM followed by 8 2-fold serial dilutions and one blank. DMSO solvent correction and double referencing were used to correct the data before fitting to a 1:1 kinetic model to obtain the binding (k_a_) and dissociation (k_d_) rates and equilibrium K_d_.

### RIPK1 expression, purification, and ligations

DNA encoding RIPK1(1–294)C34A.C127A.C233A.C240A^25^ with an N-term flagHis tag followed by TEV cleavage site and C-term alanine followed by GyrA-CBD was synthesized at Genscript and inserted into pFASTBAC1. Plasmid DNA was transformed into DH10Bac cells, the recombinant Bacmid (white colony) was grown up overnight and the recombinant Bacmid DNA was prepared using Qiagen mini prep kit. SF9 cells were transfected with Bacmid DNA using Fugene transfection reagent to make Baculovirus. Baculoviral infected insect cells were generated and protein was expressed in SF9 cells. FlagTEVRIPK1(1–294)C34A.C127A.C233A.C240AGyrA-CBD was released from Sf9 cells by sonication and captured onto chitin resin from clarified lysate supernatant. 2 mM peptides, C[K-PEG2-Biotin] or C-ln-peptide in 0.2 M MESNA in 50 mM HEPES 150 mM NaCl 10% glycerol or 50 mM DTT was added to the washed chitin resin and incubated at room temperature for 19 to 24 hr. The labeled and cleaved flagTEVRIPK1(1–294)C34A.C127A.C233A.C240A was concentrated and sized on Superdex 200.

### Transferrin receptor expression, purification, and ligations

DNA encoding soluble human Transferrin Receptor (TFR1) residues L122-F760 with a gp67 signal sequence followed by N-term 6hisTEV and C-term GyrA-CBD was synthesized at Genscript and inserted into pFASTBAC1. Plasmid DNA is transformed into DH10Bac cells, the recombinant Bacmid (white colony) is grown up overnight and the recombinant Bacmid DNA is prepped using Qiagen mini prep kit. SF9 cells are transfected with Bacmid DNA using Fugene transfection reagent to make Baculovirus. Baculoviral infected insect cells were generated and protein was expressed in SF9 cells. HisTEVTFR1(L122-F760)GyrA-CBD was captured from conditioned medium by cOmplete His-Tag resin (Roche), eluted with 250 mM imidazole after an extensive resin wash, followed by capture onto chitin resin. The chitin beads were thoroughly washed prior to the addition of 1.4 mM C[K-PEG2-Biotin] in 57 mM MESNA at pH = 7.5. Ligations progressed at room temperature for ~15 hr. Biotin labeled TRF1 released from chitin resin was dialyzed prior to sizing on Superdex 200.

### CD73 expression, purification, and ligation(s)

DNA encoding soluble CD73 truncated after residue S552^26^ and tagged on the C-term with flagAGyrA-CBD was synthesized at Genscript and inserted into pHTBV1mcs3. Plasmid DNA was transformed into DH10Bac competent cells, the recombinant Bacmid (white colony) was grown up overnight and the recombinant Bacmid DNA was prepped using Qiagen mini prep kit. SF9 cells were transfected with Bacmid DNA using Fugene transfection reagent to make P0 Baculovirus. The virus was amplified, and protein was secreted by transduced HEK293 cells. CD73flagAGyrACBD was captured by Chitin resin from the BacMam infected HEK293 conditioned medium treated with 20uM zinc sulfate. The chitin beads were thoroughly washed prior to the addition of 0.9 mM labeled di-peptide (C[K-PEG2-Biotin] or C[K-fluorescein]; 21^st^ Century Peptides) in 36 mM MESNA at pH = 7.5. Ligations progressed at room temperature for ~24 hr. The sCD73flagACK-label released from chitin resin was dialyzed and concentrated prior to sizing on Superdex 200.

### Control CD73 for NHS labeling

Human CD73(27–549)T376A^26^ was designed with human CD33 signal sequence and C-term 6His tag. The completed CD33ss-hCD73(27–549)T376A_6His was cloned into pHTBVmcs between BamH1-Hind3 sites. CD33ss-hCD73(27–549)T376A_6His was expressed in HEK293F cells in the presence of 20uM ZnSO4 through transfection. hCD73(27–549)T376A_6His was captured from 72 hour conditioned medium by HisTrap Excel column (GE Healthcare) and washed to baseline with 50 mM imidazole in 25 mM HEPES 0.1 M NaCl pH = 7.0. HiTrap Heparin HP columns (GE Healthcare) were connected in tandem with HiTrap Q HP Columns (GE Healthcare) immediately following the washed HisTrap Excel column. hCD73(27–549)T376A_6His was eluted with a linear imidazole gradient and sized onto Superdex 200. Purified hCD73(27–549)T376A_6His was chemically labeled with the NHS-RED labeling kit (Nanotemper) as per manufacturer instructions.

### Microscale thermophoresis (MST)

MicroScale Thermophoresis (MST)^27^ was done on the NHS-RED labeled sCD73 starting at 200 nM protein in 20 mM Trizma, pH 7.5 5 mM NaCl 10 mM MgCl2 0.3 mM CHAPS 0.01%BSA using Hydrophilic, Standard, & Hydrophobic capillaries and testing 20%, 40% & 80% MST at 75% LED. sCD73-flag-CK-fluorescein was analyzed at 25 nM and 50 nM protein, against serial dilution of compounds from 12 nM to 200uM using standard or hydrophobic capillaries and testing 20% or 40% MST at 75% LED. Analyzed on Monolith NT.115 instrument.

### IGD domain of aggrecan expression purification and ligation

DNA encoding aggrecan 351–430 (IGD domain) with an N-term RAGE signal sequence with an enterokinase (EK) cleavable 6His and C-term GyrA/CBD was synthesized at Genscript and inserted into pHTBV1mcs3. Plasmid DNA was transformed into DH10Bac competent cells, the recombinant Bacmid (white colony) was grown up overnight and the recombinant Bacmid DNA was prepped using Qiagen mini prep kit. SF9 cells were transfected with Bacmid DNA using Fugene transfection reagent to make P0 Baculovirus. The virus was amplified, and protein was secreted by transduced HEK293 cells. H6/EK/IGD/GyrA/CBD was captured by Chitin resin from the BacMam infected HEK293 conditioned medium supplemented with 25 mM HEPES pH = 7.5, 0.35 M NaCl 0.25%CHAPS 1 mM CaCl2. The chitin beads were thoroughly washed prior to the addition of 1 to 2 mM C[K-PEG2-Biotin in 40 to 80 mM MESNA at pH = 7.5. Ligations progressed at room temperature overnight. The H6/EK/IGD/C[K-PEG2-biotin] released from chitin resin was dialyzed, concentrated, flowed through anti-flag M2 resin (Sigma) prior to sizing on Superdex 200.

## Data Availability

The datasets generated during and/or analyzed during the current study are contained in the manuscript or are available from the corresponding author upon reasonable request
